# Evolutionary Analysis of the Melon (*Cucumis melo* L.) GH3 Gene Family and Identification of GH3 Genes Related to Fruit Growth and Development

**DOI:** 10.3390/plants12061382

**Published:** 2023-03-20

**Authors:** Sheng Chen, Kaiqin Zhong, Yongyu Li, Changhui Bai, Zhuzheng Xue, Yufen Wu

**Affiliations:** 1 Agricultural Bioresources Research Institute, Fujian Academy of Agricultural Sciences, Fuzhou 350003, China; 2 Fuzhou Institute of Vegetable Science, Fuzhou 350018, China; 3 College of Horticulture, Fujian Agriculture and Forestry University, Fuzhou 350002, China; 4 Crops Research Institute, Fujian Academy of Agricultural Sciences, Fuzhou 350013, China

**Keywords:** evolutionary analysis, expression analysis, fruit development, GH3 gene family, melon

## Abstract

The indole-3-acetic acid (IAA) auxin is an important endogenous hormone that plays a key role in the regulation of plant growth and development. In recent years, with the progression of auxin-related research, the function of the Gretchen Hagen 3 (GH3) gene has become a prominent research topic. However, studies focusing on the characteristics and functions of melon GH3 family genes are still lacking. This study presents a systematic identification of melon GH3 gene family members based on genomic data. The evolution of melon GH3 family genes was systematically analyzed by means of bioinformatics, and the expression patterns of the GH3 family genes in different melon tissues during different fruit developmental stages and with various levels of 1-naphthaleneacetic acid (NAA) induction were analyzed with transcriptomics and RT-qPCR. The melon genome contains 10 GH3 genes distributed across seven chromosomes, and most of these genes are expressed in the plasma membrane. According to evolutionary analysis and the number of GH3 family genes, these genes can be divided into three subgroups, and they have been conserved throughout the evolution of melon. The melon GH3 gene has a wide range of expression patterns across distinct tissue types, with expression generally being higher in flowers and fruit. Through promoter analysis, we found that most *cis*-acting elements contained light- and IAA-responsive elements. Based on the RNA-seq and RT-qPCR analyses, it can be speculated that *CmGH3-5*, *CmGH3-6* and *CmGH3-7* may be involved in the process of melon fruit development. In conclusion, our findings suggest that the GH3 gene family plays an important role in the development of melon fruit. This study provides an important theoretical basis for further research on the function of the GH3 gene family and the molecular mechanism underlying the development of melon fruit.

## 1. Introduction

Melon (*Cucumis melo* L.), also known by the name of the popular melon cultivar cantaloupe, is an economically important crop and an annual diploid herb of the Cucurbitaceae (cucumber) family [1,2]. In addition to being eaten as a fruit, melon also has medicinal properties, such as analgesic, anti-inflammatory, antioxidant, anticancer, and diuretic effects, as well as activities related to the prevention of hypothyroidism, atherosclerosis and other diseases [3,4,5]. The production of fruit is an important stage in the melon plant life cycle, and the development and ripening of the fruit are complex processes [6,7]. Understanding the mechanism of melon fruit development and ripening plays an important role in the picking, preservation and storage of the fruit, and can also enhance its economic value [6,7].

Auxin is an important endogenous hormone in plants and is involved in the regulation of plant growth and development [8,9]. Auxin primordial response genes include three main categories, namely, auxin/indole-3-acetic acid (Aux/IAA), small auxin upregulated RNAs (SAUR) and Gretchen Hagen 3 (GH3) [8,9]. In 1987, the first GH3 gene was found in *Glycine max*, and later, the corresponding GH3 genes were found in *Arabidopsis thaliana*, *Nicotiana tabacum* and *Oryza sativa* [10,11]. In recent years, with the advancement in auxin research, functional studies on the GH3 gene have become popular [8,9]. The GH3 protein has been found to function as an enzyme involved in the synthesis of IAA and jasmonic acid (JA) [12]. In addition, it can also interact with auxin response factors and participate in the light response pathway [12]. The GH3 protein has auxin amino acidylation activity, which has been verified in *A. thaliana* and using various auxins in vitro [13]. Most *A. thaliana* GH3 proteins have amino acid synthase activity and act on one or more auxins; specifically, JAR1 (GH3.11) can bind amino acids to JA [14]. Since the discovery of the GH3 gene, research on this gene in plants has mainly focused on *A. thaliana* and plants overexpressing *YDK1*/*GH3* [15,16]. However, most of the *A. thaliana* GH3 loss-of-function mutants that have been studied did not change in morphology, although they were sensitive to exogenous auxin [17,18]. For example, the phenotype of the *PpGH3-1* knockout mutant in *Physcomitrella patens* is basically the same as that of the wild type, which indicates that the function of the GH3 gene may have some redundancy [19].

With the development of sequencing technology, the genomes of many species have been published. This has led to great progress in the study of the GH3 gene family, and many plant GH3 gene family members have been cloned and identified [20,21]. Many studies have investigated the function of the GH3 gene family in model plants, including fruit and vegetable crop plants. In grapes, the expression of GH3 increases with the development of the fruit, significantly reducing the content of free IAA in the fruit and thereby effectively promoting fruit ripening; a similar phenomenon has also been reported in tomatoes and other crops [22,23,24]. The evolution, function and taxonomy of this gene family in melons have not been systematically studied. In this study, members of the GH3 family were systematically identified based on melon genome data, and bioinformatic analysis was performed. The chromosomal distribution, evolutionary relationships, gene duplication and promoter *cis*-acting elements of the GH3 gene were analyzed. RT-qPCR was used to analyze the expression of GH3 genes in different melon tissues during different developmental stages of the fruit and under treatment with exogenous IAA to reveal their possible biological functions. The results provide insight into the role of the GH3 gene family in plants, establishing a basis for further research on the growth and development of melon fruit.

## 2. Results

### 2.1. Identification of the Melon GH3 Gene Family

Previous studies have shown that three *Cucumis* species (*C. sativus* (2n = 2x = 14), *C. hystrix* (2n = 2x = 24) and *C. melo* (2n = 2x = 24)) were formed by differentiation of the same species and that their genomes are highly collinear and similar [25,26]. The copy number changes in the GH3 gene family during the evolution of melon were systematically assessed in this study. First, using HMMsearch and the NCBI-CDD database, the aforementioned three *Cucumis* species were found to contain 10 GH3 protein sequences, which is consistent with the known evolutionary relationship of cucumber family species (Table 1, Figure S1). We named the identified genes *CmGH3-1*~*CmGH3-10* according to the chromosomal positions of the 10 melon sequences. The length of the CmGH3 family gene open reading frame (ORF) was 1892–2604 bp. The encoded protein contained 588–640 amino acid residues, the relative molecular mass was 66,217.0–71,738.1 Da, and the theoretical isoelectric point was 5.17–7.10. The results of the subcellular localization prediction showed that nine proteins were located in the plasma membrane and that 1 protein was located in the nucleus (Table 1).

**Figure 1:**
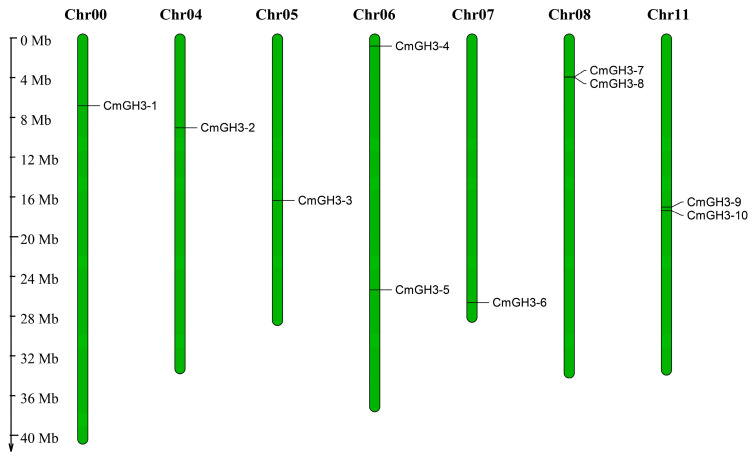
Chromosome location of the melon GH3 genes.

The ten CmGH3 genes were distributed across seven melon chromosomes (Chr00, Chr04, Chr05, Chr06, Chr07, Chr08, and Chr11; Figure 1). Among these chromosomes, Chr06, Chr08 and Chr11 were revealed by staining to contain the most (two) GH3 genes, and the remainder of the aforementioned chromosomes contained only one GH3 gene each. Although Chr00 had the greatest length, it contained only one GH3 gene. These findings suggest that there is no direct relationship between melon GH3 gene distribution and chromosome length.

### 2.2. Evolutionary Analysis of the Melon GH3 Gene

**Figure 2:**
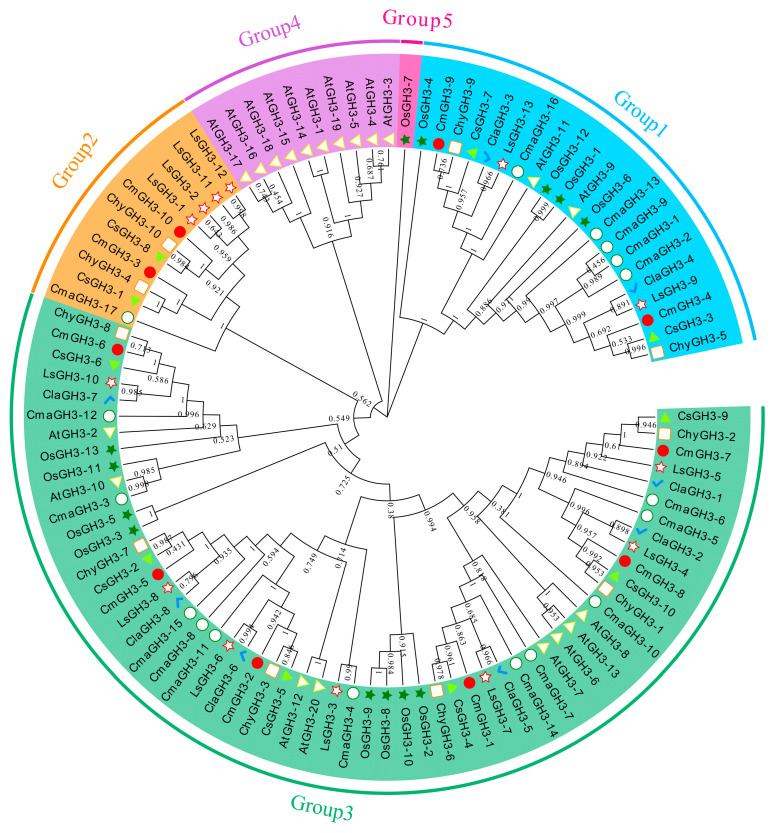
Phylogenetic relationships of the GH3 gene family in plants. The GH3 gene family was identified in three *Cucumis* species (*C. sativus*, *C. hystrix*, and *C. melo*), three additional Cucurbitaceae species (bottle gourd, watermelon and squash) and two other non-Cucurbitaceae species, *A. thaliana* and rice. The GH3 gene family belonging to a given plant species is marked with the indicated leaf label decorations. Blocks of Groups 1–5 are highlighted with sky blue, orange, green, purple and pink, respectively. The white box represents *C. hystrix* GH3 genes. The green triangle represents rice GH3 genes. The red circle represents *C. melo* GH3 genes. The green triangle represents *C. sativus* GH3 genes. The blue tick represents watermelon GH3 genes. The white five-pointed star represents bottle gourd GH3 genes. The white circle represents squash GH3 genes. The white triangle represents *A. thaliana* GH3 genes.

The study of various GH3 genes in model plants enables accurate classification of the genes and prediction of their potential role in melon. The full-length GH3 protein sequence was used to construct the phylogenetic tree for eight plant species (three *Cucumis* species—*C. sativus*, *C. hystrix*, and *C. melo*; three additional Cucurbitaceae species—bottle gourd, watermelon and squash; and two other non-Cucurbitaceae species—*A. thaliana* and rice; Figure 2 and Table S1). The evolutionary tree was divided into five groups. Groups 1, 2, and 3 contained, respectively, two, two and six melon GH3 proteins, and Group 4 and 5 did not contain melon GH3 proteins. Group 1, except for rice and bottle gourd, contained 4 and 5 GH3 sequences, the remaining species contained 2 GH3 sequences each. In Group 2, *A. thaliana* did not have GH3 sequences, rice and watermelon had no GH3 protein assigned, the remaining species each contained 1–4 GH3 sequences, and the three *Cucumis* species also contained 2 GH3 sequences each. In Group 3, *A. thaliana* and rice each contained 8 GH3 sequences, bottle gourd contained 11, and the remaining species each contained 6 GH3 sequences. Group 4 contains only 10 *A. thaliana* GH3 sequences and Group 5 contains only one rice GH3 sequence. This shows that the genes of the different groups have expanded differently in different species during the evolution of the melon GH3 gene family, and that these differences may be related to the planting environment of the crop and the ripening of the fruit. Although Group 1 and Group 2 had relatively few members, these members persisted during melon evolution, suggesting that they may have played an important role in biological processes. The number of the three *Cucumis* species genes in each subgroup was the same, consistent with melon’s evolutionary relationship.

**Figure 3:**
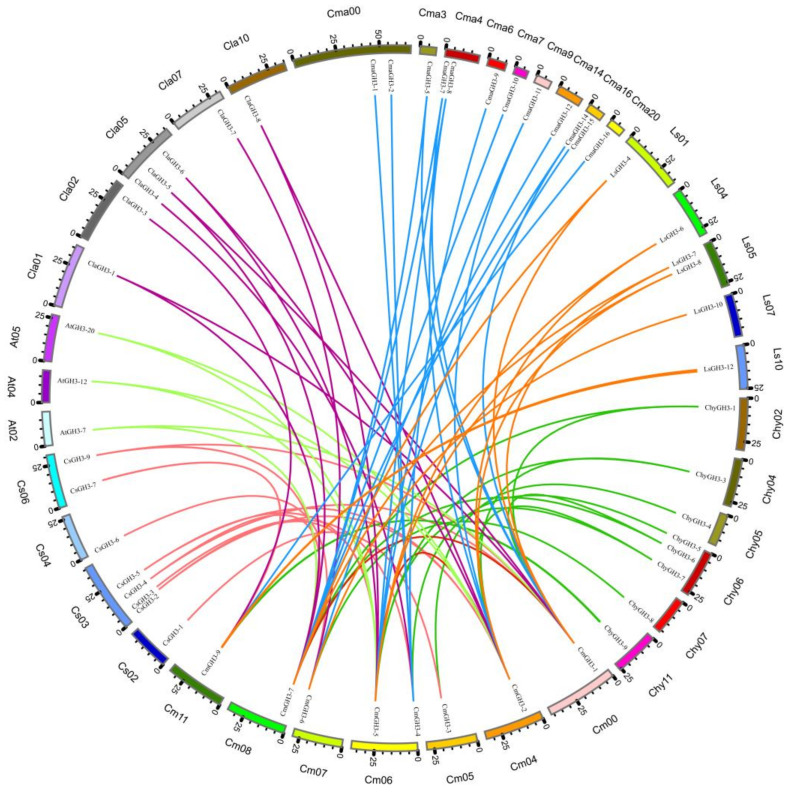
Circos diagram of the GH3 gene for the eight plant species. The collinear gene pairs in melon are connected by red lines. The collinear gene pair between melon and cucumber is connected by a pink line. The collinear gene pair between melon and *C. hystrix* is connected by a green line. The collinear gene pair between melon and bottle gourd is connected by an orange line. The collinear gene pairs between melon and watermelon are connected by purple lines. The collinear gene pair between melon and squash is connected by a blue line. The collinear gene pair between melon and *A. thaliana* is connected by a light green line.

Based on gene number, chromosome mapping, and phylogenetic tree analysis, GH3 was found to be conserved in melon evolution. To further study the evolutionary relationship of melon GH3, we selected melon as the core and constructed the collinear relationship of melon with seven species (Figure 3). We found that the GH3 family genes from melon are collinear with those of the three non-*Cucumis* Cucurbitaceae species and *A. thaliana*. However, the number of GH3 genes in *A. thaliana* is significantly higher than in melon, but with only six pairs of collinear genes, which is significantly fewer than in melon and in the three non-*Cucumis* Cucurbitaceae species. Moreover, there was no collinear gene pair for the GH3 of rice. Melon and other *Cucumis* species had the largest number of collinear gene pairs (12 pairs), and melon had 2 collinear gene pairs. To better understand the evolutionary relationship of GH3, we used OrthoFinder to construct a phylogenetic tree (Figure S2 and Table S2) for the GH3 genes of the eight species. The three *Cucumis* species were located on the same branch of the evolutionary tree, and melon was closely related to the three non-*Cucumis* Cucurbitaceae species, which is consistent with the known evolutionary relationships among the species. These combined results suggested that the melon GH3 gene family is evolutionarily conserved.

### 2.3. Evolutionary Tree, Gene Structure and Motif Analysis of the Melon GH3 Genes

**Figure 4:**
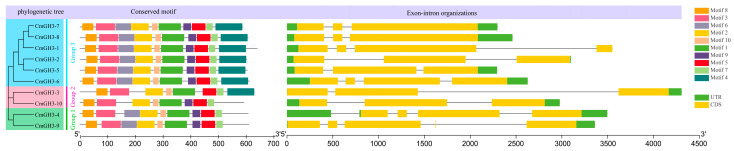
Evolutionary tree, gene structure and conservative motif analysis of the GH3 gene family in melon. Different motifs are displayed in different colored boxes as indicated on the right side, yellow indicates CDS, and gray line indicates introns.

To better understand the composition of the melon GH3 genes, we compared the different gene structures. All 10 melon GH3 genes had 2–4 introns (Figure 4). All melon GH3 members were divided into three subgroups according to the evolutionary tree results. In addition to the absence of a motif 6 structure in the GH3 gene of the Group 2 subgroup, *CmGH3-3* lacked a motif 9 structure, *CmGH3-10* lacked a motif 4 structure, and both had 2 introns. The GH3 gene in the Group 1 subgroup lacked one motif 4 structure and contained the remaining 9 motif structures. Group 3 contained all 10 motif structures. The *CmGH3-3* gene had the greatest length and contained 3 exons but only 9 motifs. The *CmGH3-5* gene had the shortest length, but it contained all motifs, indicating that the distribution of motifs in the melon GH3 genes is not highly correlated with the length of the genes.

### 2.4. Analysis of Cis-Acting Elements in the Promoters of Melon GH3 Genes

**Figure 5:**
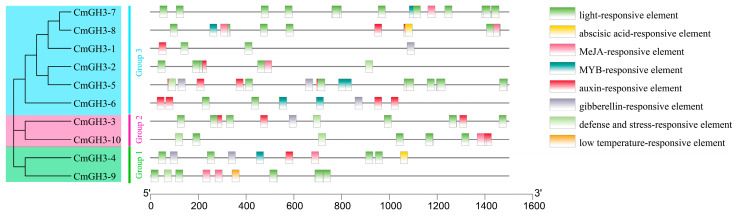
Analysis of promoter *cis*-acting elements of CmGH3 genes. *Cis*-acting elements are represented by different colored boxes.

An analysis of the *cis*-acting element in the promoter region of the melon GH3 genes revealed abundant types of promoter elements, and we found that most of the CmGH3 genes had light-responsive elements, plant hormone response elements, stress response elements, and *cis*-acting elements in the MYB binding site (Figure 5 and Table S3). Different CmGH3 genes contained different types and numbers of *cis*-acting elements, which indicated that different melon GH3 family members may exert their biological functions through different signaling pathways.

### 2.5. Bioinformatics Expression Analysis of Melon GH3 Genes

**Figure 6:**
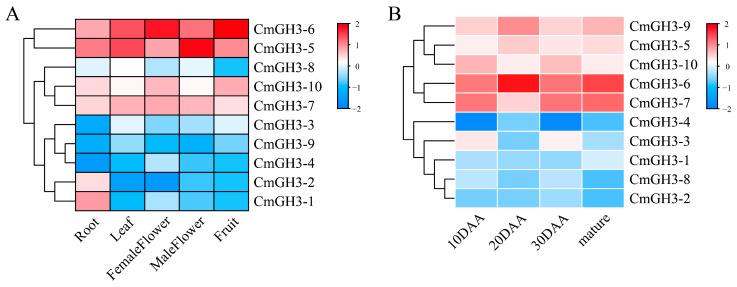
Expression analysis of GH3 genes in melon. (**A**) Tissue-specific expression analysis. (**B**) Expression analysis of melon in four developmental stages (10 DAA, 20 DAA, 30 DAA and fruit flesh).

Gene expression pattern analysis can provide important information for studying gene function. To gain insight into the expression pattern of the melon GH3 genes, we analyzed the tissue-specific expression pattern of 10 melon GH3 genes during plant growth and development (Figure 6). The results of tissue-specific analysis (Figure 6A) showed that more than half of the GH3 genes were expressed in different tissues, and the rest had no obvious tissue specificity. Among the gene family members, two genes (*CmGH3-1* and *CmGH3-2*) were highly expressed in roots, indicating that they may play an important role in melon roots. However, four genes (*CmGH3-4*, *CmGH3-5*, *CmGH3-6* and *CmGH3-10*) were more highly expressed in flowers and fruits than in other tissues.

Expression analysis of GH3 genes in melon at 10 days after anthesis (DAA), 20 DAA, 30 DAA and at the mature stage (Figure 6B) showed that the expression of more than 2/3 of the genes increased. Two GH3 genes (*CmGH3-3* and *CmGH3-10*) had the highest expression levels at 10 DAA. *CmGH3-4*, *CmGH3-5*, *CmGH3-6* and *CmGH3-9* had the highest expression levels at 20 DAA, and *CmGH3-7* had the lowest expression level at 20 DAA. *CmGH3-1*, *CmGH3-4*, *CmGH3-6* and *CmGH3-7* had the highest expression levels at the mature stage.

### 2.6. RT-qPCR Analysis of Melon GH3 Gene Expression under Exogenous NAA Treatment and during Growth and Development

**Figure 7:**
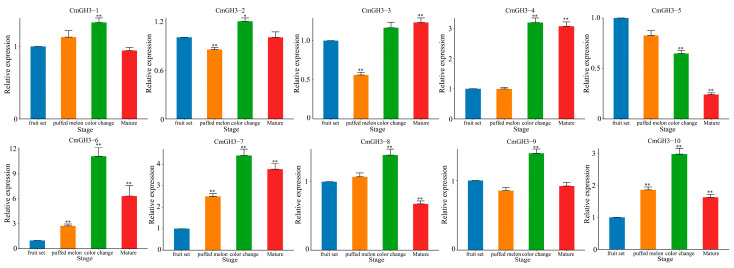
GH3 gene expression analysis in the four melon developmental stages (fruit set, swelling melon, color change and maturity). Error bars represent the average of three replicates ± SE. The difference from the control group (fruit set stage) is statistically significant: * *p* < 0.05, ** *p* < 0.01.

To further screen for candidate genes that are related to the process of melon development, we performed RT-qPCR on the CmGH3 genes at four different stages of fruit development (fruit set, swelling melon, color change, and maturity) (Figure 7). Compared with the fruit set stage, the RNA transcription levels of all of the CmGH3 genes were significantly induced at different stages, and the significant changes in expression levels indicated that these genes may be involved in the process of fruit development. Among the genes, only *CmGH3-5* was significantly downregulated in all four developmental stages, indicating that *CmGH3-5* may be a negative regulator of the development of melon fruit. Compared with the fruit set stage, although other CmGH3 genes were significantly upregulated, the expression levels of *CmGH3-4*, *CmGH3-6* and *CmGH3-7* were all upregulated more than 2-fold. This suggests that these four genes (*CmGH3-4*, *CmGH3-5*, *CmGH3-6* and *CmGH3-7*) may play a role in melon development.

**Figure 8:**
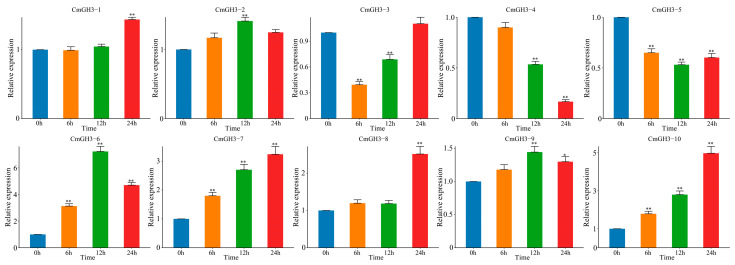
Expression analysis of the GH3 genes in melon following NAA treatment. Error bars represent the average of three replicates ± SE. The difference from the control group (NAA treatment 0 h) was statistically significant: * *p* < 0.05, ** *p* < 0.01.

To investigate the role of the melon GH3 genes in response to IAA, we analyzed the expression patterns of 10 genes following 1-naphthaleneacetic acid (NAA) treatment (Figure 8). With NAA treatment, the RNA transcription levels of all the CmGH3 genes were significantly induced in different periods, and the expression levels were significantly increased or decreased compared with the levels at 0 h. Compared with the levels at 0 h, three genes (*CmGH3-3*, *CmGH3-4* and *CmGH3-5*) were significantly downregulated. Among these genes, *CmGH3-4* and *CmGH3-5* were downregulated at all the time points; however, there was no significant difference between the expression levels of *CmGH3-3* at the 0 h and 24 h time points. The expression levels of *CmGH3-6*, *CmGH3-7* and *CmGH3-10* were significantly upregulated over time. These comprehensive results show that *CmGH3-4*, *CmGH3-5*, *CmGH3-6* and *CmGH3-7* may play an important role in melon fruit development by impacting the IAA pathway.

### 2.7. Tissue-Specific Expression of Melon GH3 Genes as Measured by RT-qPCR

**Figure 9:**
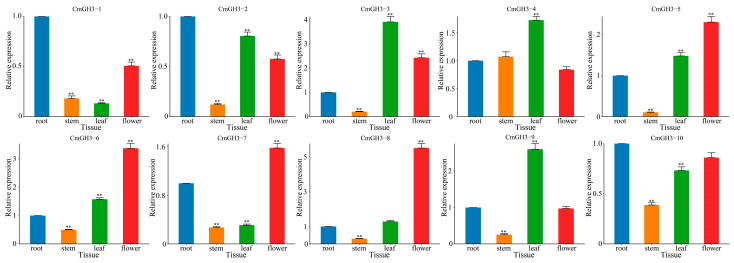
Tissue-specific expression analysis of GH3 genes in melon. Error bars represent the average of three replicates ± SE. The difference from the control group (root tissues) was statistically significant: ** *p* < 0.01.

We selected melon root, stem, leaf and flower tissues to verify the tissue expression patterns of the CmGH3 genes (Figure 9). All CmGH3 genes showed significant differential expression in different tissues. All genes except *CmGH3-4* had the lowest expression in stems, and *CmGH3-1*, *CmGH3-2*, *CmGH3-7* and *CmGH3-10* had significantly lower expression levels in leaves. However, the expression levels of *CmGH3-1*, *CmGH3-2* and *CmGH3-3* in flowers were significantly lower than the levels in roots but higher than the levels in stems and leaves. Compared with melon root tissues, *CmGH3-5*, *CmGH3-6*, *CmGH3-7* and *CmGH3-8* had significantly higher expression levels in flowers. These results showed that the expression pattern of the CmGH3 gene is complex. However, additional in-depth studies are needed to analyze its important role in melon fruit development and to provide a molecular basis to verify its function and analyze its molecular mechanism.

### 2.8. Protein Interaction Network Analysis

**Figure 10:**
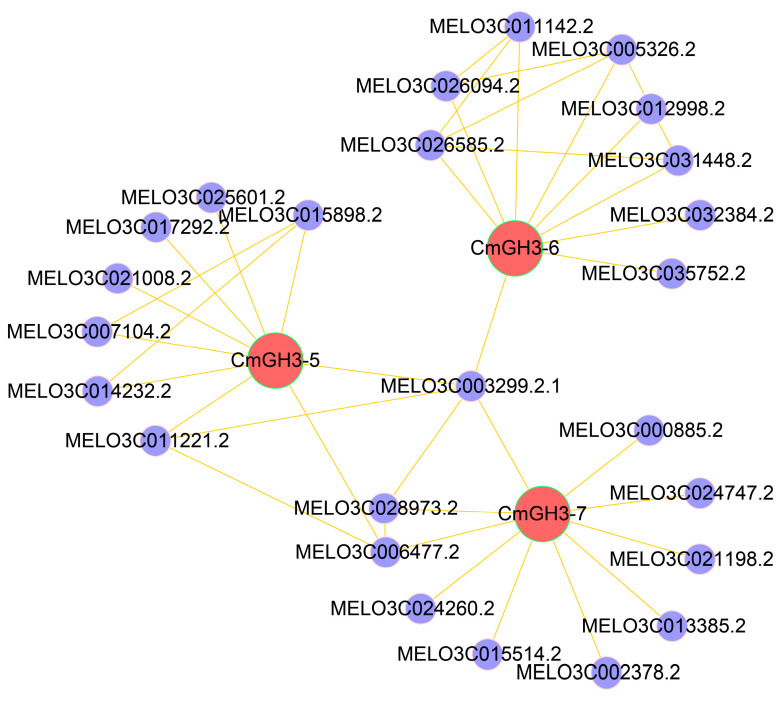
*CmGH3-5*, *CmGH3-6* and *CmGH3-7* gene interaction network.

We mapped the *CmGH3-5*, *CmGH3-6* and *CmGH3-7* gene interaction networks based on the *A. thaliana* GH3 gene (Figure 10, Figure S3). The network contains 28 nodes (genes) and 38 edges (regulatory relationships), and the above results showed that *CmGH3-5*, *CmGH3-6* and *CmGH3-7* may play an important synergistic role in regulating melon fruit growth and development. This network provides a molecular basis for further research on the functions and molecular mechanisms of the *CmGH3-5*, *CmGH3-6* and *CmGH3-7* genes in different stages of melon growth and development.

## 3. Discussion

Many studies have shown that genome-wide identification and expression analysis can help elucidate the origin, diversity, and biological function of these gene families [20,21,22,26]. In recent years, with the improvement of sequencing technology and the reduction of sequencing costs, melon genome sequencing completion provided important basic data for the mining and functional study of important melon functional gene families at the genome-wide level [27]. In the past, many studies have been devoted to the exploration of key elements related to plant development and the elucidation of their regulatory mechanisms [28]. The mining of candidate genes related to plant development is thought to play an important role in this research process [29,30]. Due to the importance of the transformation between IAA-bound and IAA-free states in plants on the regulation of plant growth and development, including fruit development and maturation, the identification and functional study of plant GH3 family genes have attracted increasing attention in recent years [8,9]. GH3 proteins are a small polygenic family (limited to plants), and in the model plant *A. thaliana*, 19 GH3 proteins are subdivided into three subfamilies. In this study, we systematically identified and performed an evolutionary analysis of melon GH3 genes. Moreover, we provided the genes and protein attributes of 10 CmGH3 proteins, the relevant evolutionary relationships among eight species, and the gene expression patterns in different tissues and at different developmental stages.

From an evolutionary point of view, the GH3 gene is prevalent in terrestrial green plants [13,14,15,16,17,18]. However, it is absent in the relatively simple algae plant *Chlamydomonas reinhardtii*, and the slightly more complex bryophyte *Physcomitrella patens* contains two GH3 genes, both of which encode enzymes that catalyze the binding of amino acids to IAA and JA [9,31]. Through evolutionary and collinear analysis, we found that there were more gene duplications and gene insertions in *A. thaliana* Group 3, and these additional GH3 genes were mostly redundant in *A. thaliana* [32]. The expansion and contraction of gene families play an important role in phenotypic adaptation during speciation [33]. Duplicate genes may enhance the metabolic pathways in which they are involved and may also acquire new functions [33]. Although there are only 10 GH3 genes in melon, which is much fewer than in most plants, the small number of melon GH3 genes has nothing to do with genome size (genome size, *C. hystrix* 289 Mb, *C. sativus* 324 Mb, *C. melo* 375 Mb, *A. thaliana* 157 Mb, *Oryza sativa* 430 Mb, *Citrullus lanatus (Thunb.) Matsum* 353 Mb). With the rapid development of comparative genomics, we can easily analyze the relationship between genes in the evolution of species, providing a basis for elucidating evolutionary history. Based on collinearity and interspecies phylogenetic tree analysis (Figure 3 and Figure S2), we found that melon and *C. hystrix* GH3 genes show good genome collinearity and that their branches of the tree are the closest, which is perfectly consistent with what is known about melon’s evolutionary relationship [25,26]. Of course, melon’s evolutionary relationship with the other three non-*Cucumis* Cucurbitaceae species is also close to that of *A. thaliana* and rice. These results suggest that melon GH3 has been conserved during melon evolution.

Transcription factors (TFs) regulate plant growth, plant development and stress resistance by regulating gene expression [34]. Our analysis of the *cis*-acting element in the promoter region of the melon GH3 gene showed that the melon GH3 gene promoter has the most *cis*-acting elements with light- and IAA-responsive elements, which indicates that the melon GH3 family members may mainly regulate the light signal and IAA pathways in plants to exert their biological function (Figure 6). Studies in *A. thaliana* found that the hypocotyls of plants overexpressing *DFL1/GH3.6* were shortened under light conditions and that the length of hypocotyls was not different from that of wild-type plants under dark conditions [35]. In addition, the expression of *YDK1/GH3.2* was downregulated under blue and far-red light conditions [15]. The overexpression of *AtGH3a/GH3.5* and the gene knockout mutant wes1 shortened and lengthened the hypocotyl, respectively, after irradiation with red light [36]. Notably, the auxin response factor (ARF) was first discovered as a protein that can specifically bind to the auxin response element of the *A. thaliana* GH3 gene [8]. The ARF regulates plant primordial response genes by specifically binding to their TGTCTC sequences [8]. The expression levels of the GH3 gene in soybean and several Aux/IAA and SAUR genes in *A. thaliana* were significantly reduced in several functionally deficient *NPH4/ARF7* gene mutants [37]. Both auxin and light are important factors affecting plant growth and development. Information on the regulation of plant GH3 genes by the light response is currently limited to those of *A. thaliana*, and there are relatively few studies on other species [38]. However, the findings for *A. thaliana* have shown that GH3 genes are involved in the regulation of the light response and even play a role in linking the auxin signaling pathway with the light response signaling pathway, which is very important in melon. Understanding the impact of the light response during fruit growth and development is of great interest.

Gene expression patterns are usually closely related to gene functions. *GhGH3.8* has been found to be highly expressed in the flowers of cotton, and 8 GH3 genes were also highly expressed in the flowers of rice [39,40]. The expression levels of *SlGH3-5*, *SlGH3-9*, *SlGH3-14* and *SlGH3-15* in tomato plants are extremely low at the fruit development stage, and the genes related to tomato ripening, *SlGH3-1* and *SlGH3-2*, are both highly expressed and involved in the fruit ripening process [41]. We found that the expression levels of *CmGH3-5*, *CmGH3-6*, *CmGH3-7* and *CmGH3-8* in melons were significantly higher in flowers than in other tissues (Figure 9). In *A. thaliana*, *AtGH3.9* can be inhibited by exogenous IAA, resulting in overall changes in promoter activity, enhanced promoter activity in lateral roots, and inhibition of MeJA-induced root growth in *AtGH3.9* mutants [42]. In pea plants, *PsGH3.5* can respond to ABA, IAA, GA3, MeJA, SA, etc., and can be significantly induced under different light conditions and after treatment with auxin herbicides [43]. Previous studies have shown that during the growth and development of plants, the content of the hormone IAA increases or decreases. In grapes and tomatoes, IAA levels decreased significantly as fruits matured, and in melon, *CmGH3-4* and *CmGH3-5* were downregulated by IAA and upregulated by *CmGH3-6*, *CmGH3-7* and *CmGH3-10* (Figure 8). This suggests that *CmGH3-6*, *CmGH3-7* and *CmGH3-10* may be positive regulators of IAA signal transduction and that *CmGH3-4* and *CmGH3-5* are negative regulators. With the development of melon fruit, the expression of *CmGH3-5* was downregulated, and the expression of *CmGH3-4*, *CmGH3-6* and *CmGH3-7* was upregulated by greater than 2-fold (Figure 7). Tissue expression pattern analysis of genes can provide important information for studying gene function, and tissue-based expression analysis revealed that *CmGH3-5*, *CmGH3-6* and *CmGH3-7* were the most highly expressed in flowers (Figure 9). Based on these results, we speculate that *CmGH3-5* may be negative regulators of melon fruit development and that *CmGH3-6* and *CmGH3-7* are positive regulators. This provides an important clue concerning the regulation of GH3 expression by reliance on the IAA pathway during melon fruit development and is also expected to provide candidate genes for genetic breeding research with applications in melon fruit development.

Studies on some plants have shown that most processes related to plant growth, development, and physiology are regulated by networks of protein-protein interactions [27]. Reconstructing interaction networks between proteins is important for understanding how proteins function in biological systems [44]. It is important to understand the response mechanism of plant growth and development, biological signals and energy metabolism in specific physiological states, as well as the functional connection between proteins [44]. We mapped the *CmGH3-5*, *CmGH3-6* and *CmGH3-7* gene interaction networks based on the *A. thaliana* GH3 gene (Figure 10). This network provides a molecular basis for further research on the functions and molecular mechanisms of the *CmGH3-5*, *CmGH3-6* and *CmGH3-7* genes in different stages of melon growth and development.

In conclusion, we showed that genes of the GH3 family may play an important role in melon fruit development by analyzing them during different melon tissue and fruit development stages and through NAA-induced expression analysis. Based on previous NAA induction and RT-qPCR data collected during growth and development, we speculated that *CmGH3-5*, *CmGH3-6* and *CmGH3-7* may play an important role in melon fruit development. This study is the first systematic evolutionary analysis of the melon GH3 gene family and provides new insights into the differential expression of GH3 genes during melon fruit development. The findings of this study lay the foundation for further functional verification of the melon GH3 gene and the analysis of its molecular mechanism involved in fruit development.

## 4. Materials and Methods

### 4.1. Plant Material

In this study, a 50-hole plug tray (28 cm × 54 cm) was used to raise seedlings at the Dongzhang Experimental Base, Fuqing City, Fujian Province, China, and ‘Jiatian’ melon seeds were sown on 15 April 2022. Six soil ridges were built in the shed with grow lights, the width of each ridge (including the furrow) was 1.28 m and the width of each ridge surface was 0.78 m. One row was planted in each ridge, and the plant spacing was 40 cm. Greenhouse hanging vine cultivation and single vine pruning were adopted as cultivation protocols, leaving 1 to 2 fruits per vine. 1-Naphthaleneacetic acid (NAA) is a plant growth regulator with auxin activity, and Chen et al. found that exogenous NAA-treated leaves increased giant pumpkin fruit size. Roots, stems, leaves and flowers were therefore sampled at full bloom on 6 July 2022, and the leaves were sprayed with NAA (concentrations: 50 μmol/L) at 0 h, 6 h, 12 h and 24 h. Leaf samples were taken at different time points after NAA spraying. Then, fruit samples (3 biological replicates sampled every 10 days) were collected according to the developmental stage of the melon, and the samples were quickly placed into liquid nitrogen for quick freezing, transported to the laboratory, and stored at −80 °C.

### 4.2. Identification and Bioinformatics Analysis of the CmGH3 Genes

The latest published genome sequence was used as a reference in this study [45]. The genomic and proteomic data of melon, cucumber, *C. hystrix*, bottle gourd, watermelon and squash were downloaded from the CuGenDB database (http://cucurbitgenomics.org/, accessed on 10 May 2022). The *A. thaliana* genome and protein sequences were downloaded from the *A. thaliana* Genome Database on The Arabidopsis Information Resource (TAIR) website (https://www.arabidopsis.org/, accessed on 10 May 2022) and from the Rice Genome Database (http://rice.plantbiology.msu.edu/, accessed on 10 May 2022). The hidden Markov model of the GH3 gene domain (PF03321) and the hidden Markov model (HMM) in HMMER software (http://hmmer.org/, accessed on 10 May 2022) were used to identify melon, cucumber, *C. hystrix*, bottle gourd, and protein sequences in watermelon and squash [46]. Then, all candidate genes were validated in the NCBI-CDD (https://www.ncbi.nlm.nih.gov/cdd/, accessed on 10 May 2022) database [47]. ExPASy software (http://cn.expasy.org/tools, accessed on 11 May 2022) was used to calculate the number of amino acid residues, relative molecular mass, and theoretical isoelectric point of the CmGH3 protein, and EuLoc software (http://euloc.mbc.nctu.edu.tw/, accessed on 11 May 2022) was used to predict the subcellular localization of the CmGH3 protein [48].

### 4.3. Phylogenetic and Collinearity Analysis of the CmGH3 Gene Family

The GH3 protein sequences of *A. thaliana*, rice, melon, cucumber, *C. hystrix*, bottle gourd, watermelon and squash were used for multiple sequence alignment using Clustal W in MEGA 7 software. Then, based on the alignment results, the proximity method was used to construct a phylogenetic tree with a bootstrap value set to 1000 [49]. The resulting phylogenetic tree was designed with the online tool EvolView (https://evolgenius.info/, accessed on 12 May 2022). A Basic Local Alignment Search Tool (BLAST) analysis was performed using the GH3 protein sequences of *A. thaliana*, rice, melon, cucumber, *C. hystrix*, bottle gourd, watermelon and squash. The e value was set to 1 × 10^−10^, and the comparison results were screened and analyzed by MCScanX software. The drawings were generated using Circos software.

### 4.4. Chromosomal Location, Gene Structure and Motif Analysis of the CmGH3 Gene Family

The chromosomal location information of CmGH3 gene family members was extracted by TBtools software, and the chromosomal location map of the GH3 genes was drawn with MapChart software. The phylogenetic tree of CmGH3 was constructed with MEGA 9 software, and the nwk file was obtained. Motif analysis was performed by the MEME program (with the number of functional domains set to 10). All the above results were visualized using TBtools software [50,51].

### 4.5. Analysis of Upstream Cis-Acting Elements of the CmGH3 Genes

According to the position information of the CmGH3 genes, the upstream 1500 bp DNA sequence was extracted as the promoter sequence, and the PlantCARE database (http://bioinformatics.psb.ugent.be/webtools/plantcare/html/, accessed on 12 May 2022) was used to predict the possible *cis*-acting elements. Tbtools software was used for visualization [51,52].

### 4.6. RNA-Seq Analysis

From the CuGenDB database (http://cucurbitgenomics.org/, accessed on 14 May 2022), RNA-seq data for melon tissue (roots, stems, leaves and flowers) and growth stages (melon fruit flesh at 10 days after anthesis (DAA), 20 DAA, and 30 DAA and fruit flesh at the mature stage) were downloaded. Fastp software was used for filtering, removing adapters, and quality control of the raw data. Hisat2 software aligned the filtered data to the melon reference gene, and StringTie software was used for expression quantification [53,54]. Gene expression was assessed using the fragments per kilobase of transcript per million mapped reads (FPKM) method, and the expression data were normalized to log10 (FPKM + 1). The expression heatmap was drawn with TBtools software [51].

### 4.7. RT-qPCR

Primer 6.0 software was used to design specific primers for the CmGH3 gene sequence (Table S4) [55]. cDNA from the 82-day-old root and leaf tissue was used as a template, and the expression of candidate genes was measured by RT-qPCR. Reverse transcription was performed using an M-MLV RTase cDNA Synthesis Kit (TaKaRa, Kyoto, Japan). 0.5 μg RNA plus 2 μL 4× DN Master Mix (with gDNA Remover added) and 8 μL RNase-free ddH_2_O were mixed thoroughly and incubated for 5 min at 37 °C for genomic DNA removal (Tiangen, Beijing, China). 2 uL RNA plus 4 μL All-In-One 5× RT MasterMix and 14 μL of nuclease-free H_2_O were mixed thoroughly under the following conditions: 37 °C for 15 min, 60 °C for 10 min, 95 °C for 3 min (TransGen Biotech, Beijing, China). The cDNA stock solution was subjected to qPCR, and the CT value was obtained. The cDNA concentration was 300 ng for subsequent RT-qPCR. RT-qPCR was performed under the following conditions: 94 °C for 15 s, followed by 38 cycles of 94 °C for 15 s, 55 °C for 15 s and 72 °C for 15 s. Relative quantification was performed using the 2^−ΔΔCt^ method [56]. Microsoft Excel 2010 software was used to process the raw data, and t tests (* and ** indicate significant differences at the *p* < 0.05 and *p* < 0.01 probability levels, respectively) were used to analyze the difference salience, and the R language ggplot2 software package was used for graphical visualization.

### 4.8. Protein Interaction Network Analysis

Through homologous protein analysis, CmGH3 gene alignment was performed on the official *A. thaliana* website (https://www.arabidopsis.org/, accessed on 14 May 2022), and CmGH3 gene orthologs were screened in *A. thaliana* and submitted to the Search Tool for the Retrieval of Interacting Genes/Proteins (STRING) (https://string-db.org/, accessed on 14 May 2022) database. The relationships within the *A. thaliana* protein interaction network were further obtained. Then, the interacting genes of *A. thaliana* were compared with the melon genome, and the orthologous genes of melon were screened. Finally, Cytoscape software was used for visualization [57].

## 5. Conclusions

In this study, genome-wide identification of melon GH3 genes was performed, and 10 GH3 genes were identified in the melon genome—a quantity that is significantly fewer than those identified in *A. thaliana* and rice. The results of the phylogenetic tree and collinearity analysis indicated that CmGH3 gene family members can be divided into three large subgroups, which have been conserved throughout the evolution of melon. Expression analysis revealed that *CmGH3-5*, *CmGH3-6* and *CmGH3-7* are important regulatory genes during melon fruit development. This study is the first to systematically analyze the evolution of the melon GH3 gene family and provides a new understanding of melon fruit development, laying a foundation for further in-depth functional analysis and the study of potential breeding applications for the CmGH3 gene family.

## Figures and Tables

**Table 1:** Characteristics of the GH3 gene family in the melon genome.

Gene ID	Gene Name	Chromosome	Open Reading Frame/bp	Protein Length/aa	Relative Molecular Weight (r)/Da	Theoretical Isoelectric Point (pI)	Subcellular Localization
MELO3C027346.2	CmGH3-1	chr00	2048	640	71,738.1	5.17	Plasma Membrane
MELO3C018166.2	CmGH3-2	chr04	1892	602	68,097.4	7.10	Plasma Membrane
MELO3C008672.2	CmGH3-3	chr05	2037	630	71,005.3	5.65	Plasma Membrane
MELO3C006046.2	CmGH3-4	chr06	2604	607	68,048.5	6.57	Plasma Membrane
MELO3C016616.2	CmGH3-5	chr06	2110	604	68,036.1	5.90	Plasma Membrane
MELO3C017825.2	CmGH3-6	chr07	2315	612	69,791.0	6.26	Nucleus
MELO3C007596.2	CmGH3-7	chr08	2094	588	66,217.0	5.73	Plasma Membrane
MELO3C007597.2	CmGH3-8	chr08	2283	607	68,630.4	5.22	Plasma Membrane
MELO3C013558.2	CmGH3-9	chr11	2043	610	68,724.8	6.02	Plasma Membrane
MELO3C013566.2	CmGH3-10	chr11	2080	592	67,741.0	6.69	Plasma Membrane

## Data Availability

The genome databases were downloaded from CuGenDB (http://cucurbitgenomics.org/, accessed on 14 May 2022). The RNA-seq datasets were downloaded from the NCBI Sequence Read Archive under accession numbers PRJNA286120 and PRJNA383830. The datasets supporting the conclusions of this article are included in the article and its Supplementary Materials.

## Supplementary Materials

The following supporting information can be downloaded at: https://www.mdpi.com/article/10.3390/plants12061382/s1, Figure S1. Distribution of the GH3 domain in the GH3 proteins of melon. Figure S2. Phylogenetic relationships of the GH3 gene family of the 8 selected species. Figure S3. *A. thaliana* GH3 protein interaction network. Table S1. GH3 gene protein sequences in this study. Table S2. Ka/Ks ratios of repeated GH3 genes. Table S3. *cis*-acting elements analysis of GH3 family members in melon. Table S4. All primers used in this study.
