# Supplementary figures and images for: Evolutionary Analysis of the Melon (Cucumis melo L.) GH3 Gene Family and Identification of GH3 Genes Related to Fruit Growth and Development
Source: Plants (Basel). 2023 Mar 20;12(6):1382. doi: 10.3390/plants12061382 (PMC10053650; doi:10.3390/plants12061382)

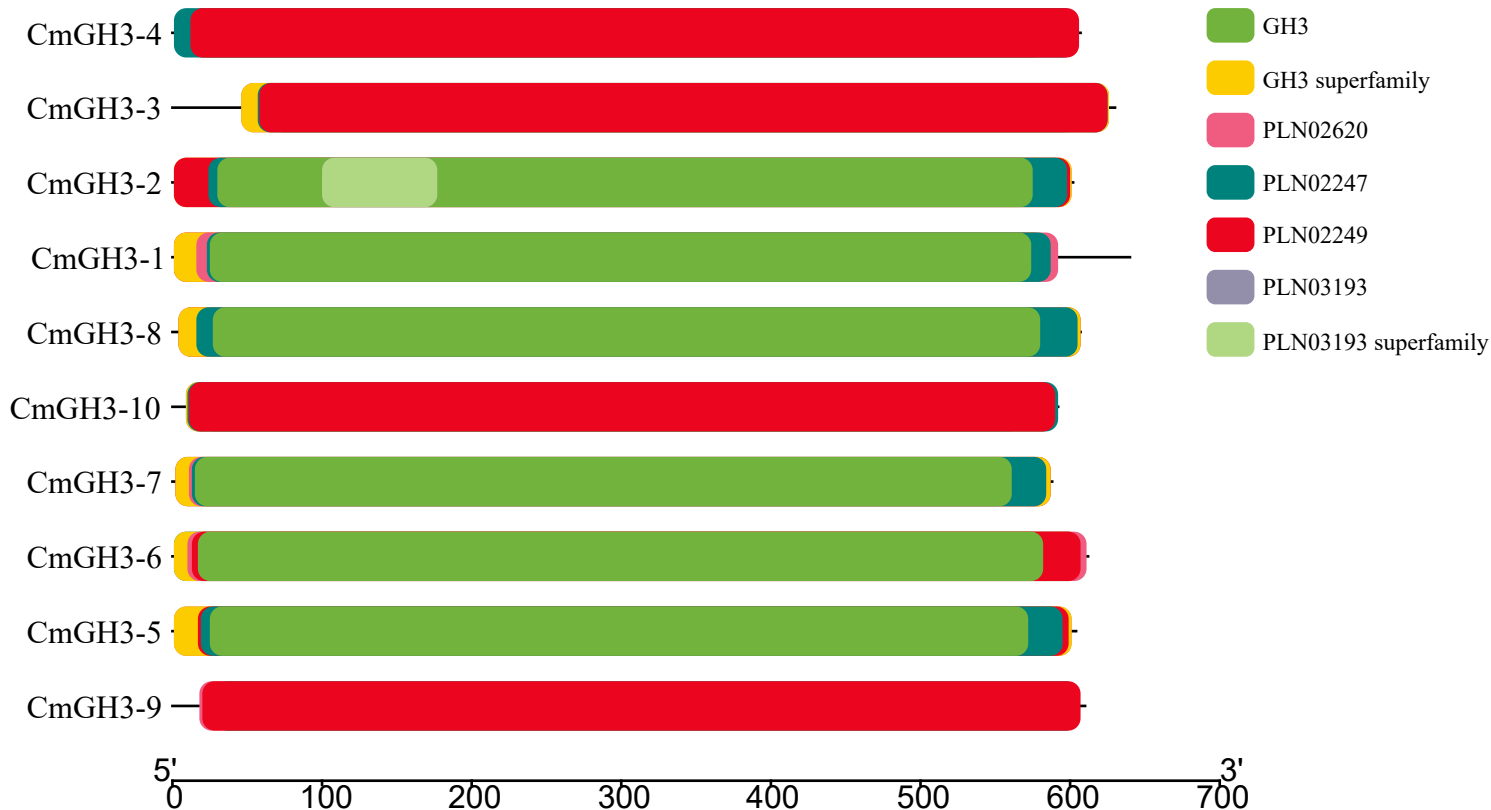

Supplement: Supplementary file 1 [file plants-12-01382-s001.zip › Supplementary Files/Fig S1.pdf]

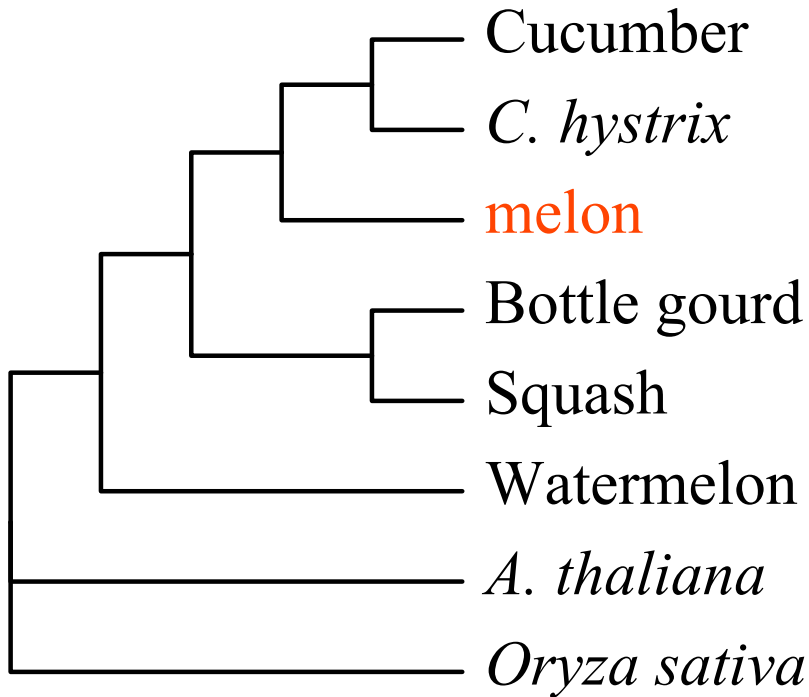

Supplement: Supplementary file 1 [file plants-12-01382-s001.zip › Supplementary Files/Fig S2.pdf]

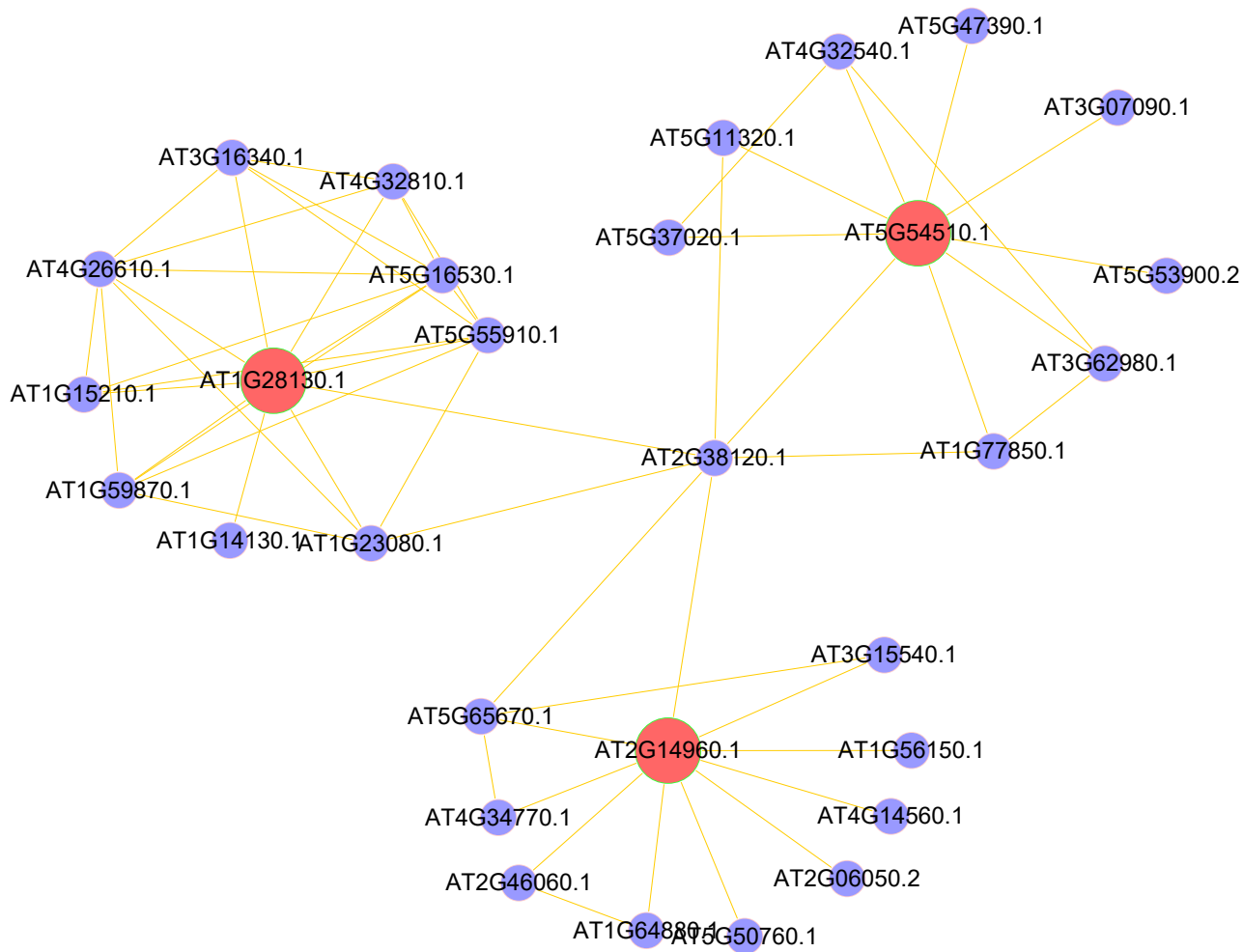

Supplement: Supplementary file 1 [file plants-12-01382-s001.zip › Supplementary Files/Fig S3.pdf]
